# Feasibility of Collecting Vulvar Pain Variability and Its Correlates Using Prospective Collection with Smartphones

**DOI:** 10.1155/2014/659863

**Published:** 2014-06-10

**Authors:** Ruby H. N. Nguyen, Rachael M. Turner, Jared Sieling, David A. Williams, James S. Hodges, Bernard L. Harlow

**Affiliations:** ^1^Division of Epidemiology & Community Health, School of Public Health, University of Minnesota, 1300 South 2nd Street, Suite 300, Minneapolis, MN 55454, USA; ^2^MEI Research, Ltd., St. Louis Park, MN 55416, USA; ^3^Chronic Pain and Fatigue Research Center, Department of Anesthesiology, University of Michigan, Ann Arbor, MI 48106, USA; ^4^Division of Biostatistics, School of Public Health, University of Minnesota, Minneapolis, MN 55455, USA

## Abstract

*Context*. Vulvar pain level may fluctuate in women with vulvodynia even in the absence of therapy; however, there is little evidence suggesting which factors may be associated with variability. *Objective*. Determine the feasibility of using smartphones to collect prospective data on vulvar pain and factors that may influence vulvar pain level. *Methods*. 24 clinically confirmed women were enrolled from a population-based study and asked to answer five questions using their smartphones each week for one month. Questions assessed vulvar pain level (0–10), presence of pain upon wakening, pain elsewhere in their body, treatment use, and intercourse. *Results*. Women completed 100% of their scheduled surveys, with acceptability measures highly endorsed. Vulvar pain ratings had a standard deviation within women of 1.6, with greater variation on average among those with higher average pain levels (*P* < 0.001). On the weeks when a woman reported waking with pain, her vulvar pain level was higher by 1.82 on average (*P* < 0.001). Overall, average vulvar pain level was not significantly associated with the frequency of reporting other body pains (*P* = 0.64). *Conclusion*. Our smartphone tracking system promoted excellent compliance with weekly tracking of factors that are otherwise difficult to recall, some of which were highly associated with vulvar pain level.

## 1. Introduction

Chronic vulvar pain affects approximately 8% of the female population under 40 years old in the USA [1], with prevalence increasing to 18% across the lifespan [2]. Pharmaceutical therapy for vulvar pain has been found to be only moderately effective, with combined therapies including physical, psychological, and alternative therapies eliciting the greatest relief [3]. Among women with severe localized pain, vestibulectomy may be an effective measure for reducing vulvar pain intensity; however, pain reduction rates with this surgery have not been found to be different from medical management [4]. In the absence of cures for vulvar pain, the vast majority of women with chronic vulvar pain employ palliative pain measures [5].

Although it is chronic, some women describe their vulvar pain as intermittent or episodic [2]. The intensity of vulvar pain has also been known to fluctuate even in the absence of treatment [6]. Most of the literature reporting changes in vulvar pain level has been from clinical trials measuring the effectiveness of a new therapy and thus tends to compare pain levels between distant times and misses weekly or even daily fluctuation in pain intensity. However, clinical trials generally select women who have sought care and therefore may not reflect the majority of women with chronic vulvar pain who have symptoms but who do not seek care for their pain [7, 8].

In addition, studies of vulvar pain variability may be limited by the use of long recall periods in estimating women's pain intensity over time [9–11]; such reports can be highly variable within and between women and can lead to concerning levels of misclassification [12, 13], while previous studies have also failed to elucidate factors that may cause either improvement or worsening of vulvar pain. An example of a potentially important factor is the presence of other comorbid pain conditions that are common among women with vulvodynia [14, 15].

Using novel smartphone technology, we first sought to describe the variability of vulvar pain level in a small cohort of women with clinically confirmed vulvodynia enrolled from an existing community-based study. Secondly, we sought to describe whether other pain-related and behavioral variables were associated with vulvar pain level and its variability.

## 2. Methods

### 2.1. Source Population

This study was approved by the Fairview-University of Minnesota Institutional Review Board and all participants provided written consent prior to participation in the study. 164 women previously enrolled in an existing population-based study on the etiology of vulvodynia were informed about the present study and its focus on monitoring of symptoms. Participants were clinically confirmed as having vulvodynia by the clinical study staff and had indicated an interest in being contacted for future women's health studies.

### 2.2. Eligibility

All women from the larger study were between the ages of 18 and 40 years at first visit and were from a large healthcare administrative database. Details of the larger study can be found elsewhere [1]. Of these, 74 of the women contacted the study staff regarding participation.

Women must have had an Android smartphone that was not on one of the major cellular network carriers because these carriers do not allow nonmarket applications to be used on their network. Women consented to providing health-related data through their own personal smartphone. Written consent was necessary and was provided at the first study visit. The primary reason for ineligibility was not meeting the technological requirements of the study (*n* = 32 were ineligible to participate).

After learning about the study's requirements, 9 women were not interested or unable to participate within the 2-month time frame, and additional 3 others were scheduled and could not reschedule before the end of recruitment, while 6 made contact after the recruitment ended. Thus, 24 women were enrolled into our study.

### 2.3. Smartphone System

Data was captured using the ActiPal EMA Android application [16] installed on smartphones carried by the participants. The application interfaced wirelessly with the PiLR Healthware server system for configuration and data storage. The smartphone application delivered notifications to the participants (ringtones or vibration), signaling them to respond to a survey through the touch screen device. The notification schedule was preconfigured by the investigator through the PiLR Healthware website and was downloaded to the participant device at initial deployment along with the survey questions. This system allowed for notifications and surveys to be delivered even when wireless data connections were not available to the device. The survey responses were uploaded from the participant devices immediately after the surveys were completed, or as soon as a data connection became available. Data was anonymized by a participant identification number on the device and therefore no telephone numbers were sent with the data; data were uploaded to the server using the encrypted HTTPS protocol. The server was housed at the academic institution of the investigators. Investigators and study staff could view and manage the uploaded deidentified data in real time through the web site and respond to technical or noncompliance issues immediately. Only the study coordinator had access to the link between the deidentified data and the participant's name and contact information.

### 2.4. Study Procedure

During June 2013, eligibility screening was performed over electronic mail to determine whether a woman had access to a compatible smartphone. If interested in the study, women were asked to attend an in-person visit to learn more about the study, provide consent, and allow the study staff to download the smartphone application to their phones. Study staff educated participants regarding submitting answers through the smartphone and resyncing their device should they be instructed to do so over the course of the study. In addition, women were informed about the frequency and nature of the prompts generated by the application to initially indicate, or remind, a woman that she was due for a survey.

Surveys were delivered the same day of the week for each week. However, notifications to indicate a survey's availability for each woman occurred at random times throughout that day. Notifications automatically appeared on the participant's phone when delivered. By selecting the notification, participants were automatically directed to their weekly survey. If a survey was not completed within 15 minutes, an automatic reminder prompt would appear with the same link to the woman's survey; these reminder prompts would continue to be sent until either the woman completed her survey or a total of eight prompts were sent. This notification method is considered a native Android interaction, adhering to recommended best practices, so receiving and responding to the notifications was familiar to Android smartphone users. Women were considered to be late on their weekly response if their survey was not completed by midnight of the day the prompt was received. If a woman had not completed her survey on the day of the week it was required, she would not be able to complete that week's survey unless she contacted the study staff who were able to override the restriction. If the study staff observed that there was a missing prompt or survey completion, they contacted the participant to provide a survey. If there was a missing prompt or survey, after contact by the study staff, all participants completed their survey within 24 hours.

### 2.5. Weekly Assessment of Vulvar Pain Level and Covariates

We chose average pain reported one time per week. Previous studies have found that recalled pain averaged over the week is nearly as useful as momentary data collected through electronic diaries [17]. Women were asked to answer the following questions regarding their experience over the last week. (1) What was your vulvar pain level from 0 to 10? (2) Did you treat your vulvar pain? (3) Did you have pain elsewhere in your body? (4) Did you wake up with vulvar pain? (5) Have you had sexual intercourse? Our technology was able to modify weekly questions on the application without having physical contact with the participants' smartphones.

### 2.6. Follow-Up Survey at the Completion of 1 Month of Tracking Vulvar Pain

At the completion of the four-week tracking phase, all participants were invited to complete an online survey regarding their opinions and experiences of the application and the study procedure. One participant did not complete this survey, leaving 23 follow-up surveys.

### 2.7. Statistical Analysis

Cross-sectional analyses comparing women according to the frequency with which they reported treatment, comorbid pain, pain upon waking, and intercourse used one-way analysis of variance. Significance tests are tests of the null hypothesis that the groups defined by frequency of report do not differ. Longitudinal analyses comparing surveys that reported versus those that did not report treatment, comorbid pain, pain on waking, or intercourse used a mixed linear model (with the restricted likelihood method); the random effect was participant and the fixed effect was report versus nonreport. Cross-sectional and longitudinal analyses were computed using JMP (v. 10.0.0 Pro, SAS Institute Inc., Cary, NC). Alternative analyses using autoregressive errors within woman had worse fit according to the Akaike information criterion (AIC) and Bayesian information criterion (BIC, also called Schwarz criterion) and are not reported. Adjusted averages and standard errors are least-squares means and associated standard errors, as computed by SAS's MIXED procedure (SAS Institute Inc., Cary, NC).

## 3. Results

A total of 24 women were enrolled in the study.  Table 1 describes the women enrolled in this study. The average age was 30 years, most were single, and three-quarters had obtained a bachelor's degree or more education. Nearly one-quarter had primary vulvodynia, defined as vulvar pain with first intercourse or first tampon use, and 52% were diagnosed with pain throughout the vestibule. Approximately half of the women had not sought care for their vulvar pain prior to enrollment into our study.

All women completed all 4 weekly surveys. For statistical analyses, 96 observations were used, comprising 4 weekly surveys from each of the 24 participants. Using the time and date stamps recorded through the smartphones, we determined that 42% of the prompts resulted initiating a survey within 10 minutes; the median was 15 minutes and the 90th percentile was 125 minutes. 90% of the weekly five-question surveys were submitted less than 1 minute after initiating the survey.

**Figure 1:**
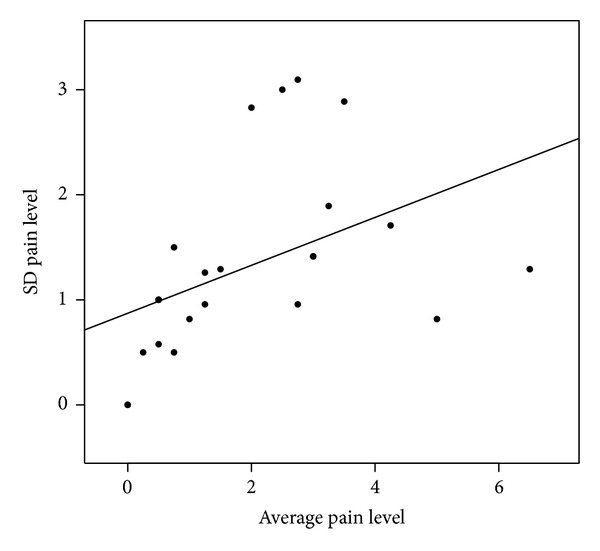
Variability, reported as standard deviation of vulvar pain report, versus average pain level for each woman.

Overall, vulvar pain ratings within women had a standard deviation of 1.58, but this variation was associated with average score.  Figure 1 plots the association between each woman's variability in her vulvar pain score (standard deviation of scores) against her average pain score. On average, the higher the mean pain level the greater the variability in her pain reports (*P* = 0.03); however, there was considerable variation in the midrange average values for pain level (i.e., scores of 2-3). There was no indication that overall ratings in this observational study tended to change with the completion of more surveys (*P* = 0.36) (results not shown).

**Figure 2:**
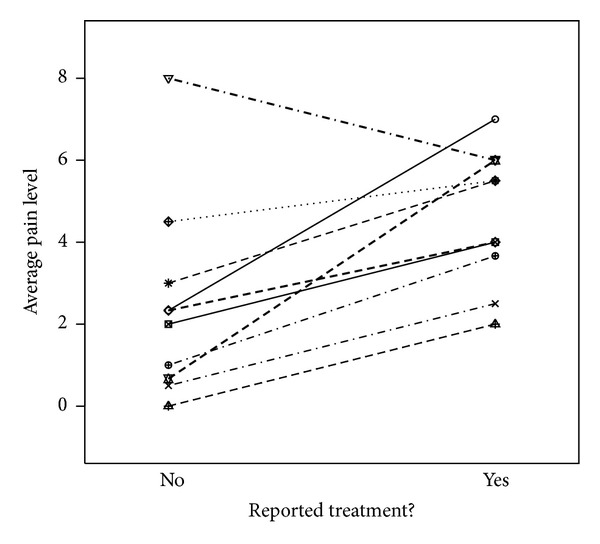
Eleven women reported receiving treatment for pain in at least one survey and reported not receiving treatment in at least one survey. For these 11 women, this figure shows the average pain scores in weeks in which treatment was used or not.

Figure 2 illustrates the differences in average vulvar pain for each of the 11 women who reported weeks with no therapy use and weeks with therapy use; average pain level was higher in weeks with reported therapy use for all women but one woman.

**Figure 3:**
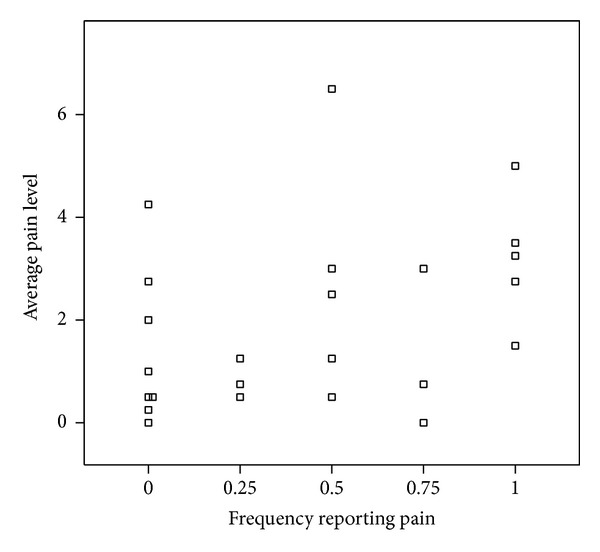
Average vulvar pain level for 24 women grouped according to frequency of reported pain elsewhere in their body.

We examined whether vulvar pain was associated with comorbid pain in two similar analyses. First, there was no trend with increasing proportion of reports of pain elsewhere in the body (*P* = 0.17). However, there may have been indication of increased average vulvar pain level among women who always had pain elsewhere on their bodies (100%) versus those who never had pain (0%) elsewhere on their bodies (Figure 3). Comparing weeks in which comorbid pain was reported to weeks in which it was not reported, weeks with a report of other pains had only slightly higher average pain score (2.09 versus 1.87, *P* = 0.64) (Table 2), whereas the average pain score was doubled in weeks in which a woman reported that she woke with pain versus weeks when she did not (3.45 versus 1.63, *P* < 0.001) (Table 2).

Table 3 describes the opinions of the participants at the end of the study regarding their experiences and their general thoughts regarding tracking. Although the women were compliant for all smartphone surveys, one woman did not complete the online feasibility survey. 96% reported that the month-long tracking of vulvar pain did not make their pain worse; however, 1 woman reported that it did. Overall, the women were a little more concerned about the effects of future tracking on vulvar pain, with 26% of women concerned or somewhat concerned that future tracking would make their pain worse. Before the study, nearly half (48%) of women did not record any of the items we queried; among those who did, they were most likely to have recorded details about their diet. However, by the end of the study, approximately half of the women reported an advantage of recording each of the time-varying factors that were studied (e.g., vulvar pain, treatment, intercourse, and mood).

Regarding the technology's feasibility, overwhelmingly and nearly uniformly, the women believed that the technology was easy to use and they liked the format of receiving prompts to complete a survey. All the women reported that they would track their pain over an extended period of time in the future. Regarding the smartphone modality itself, all but one woman indicated that they would prefer using the smartphone over a computer or paper and pencil method (Table 3).

## 4. Discussion

We studied a sample of women with clinically confirmed vulvodynia who generally did not track health-related issues before this study. Using a new smartphone modality for symptom monitoring, we found that women were highly accepting the method, which led to a 100% adherence over one month. There were few feasibility concerns with the technology, and all women were willing to continue tracking these factors in the future.

Long-term recall of time-varying factors, such as those studied here, can be subject to misclassification and potential bias. Our findings suggest that collection of data reliant upon only short-term recall of less than one week for vulvar pain, therapy use, presence of other pains, and intercourse is feasible and acceptable to women with chronic vulvar pain. Validly determining whether these factors affect remission among women with vulvodynia could profoundly influence the measurement of efficacy and effectiveness of future interventions.

The four time-varying factors that we chose to study provided additional insight into what might trigger higher vulvar pain reports. First, women who reported using treatment more frequently reported higher pain scores. This finding is not surprising, and, in fact, cross-sectional or retrospective studies cannot differentiate the temporal association between a high pain level that requires treatment initiation versus the reverse, which can lead to mistaken conclusions about treatment effectiveness.

Two-thirds of the women in our study experienced pain elsewhere on their bodies at some point during the one-month study period. However, our results regarding whether vulvar pain was associated with other body pains were not statistically significant and should be further studied with a sample size that is adequately powered to detect potentially small changes in vulvar pain level in the presence of comorbid pain. When women reported that they awoke with pain in the morning, they also reported significantly higher vulvar pain compared to the reports from other days in which they did not wake with pain, and women who reported waking with pain more frequently had higher average vulvar pain score.

We did not hypothesize that there would be an association between coital frequency and increased pain. In fact, we may have expected to see that women with lower overall pain levels were more likely to engage in sexual activity. While we found no association in either direction, there is sufficient evidence to suggest that recalled coital frequencies may be inaccurate [18] and therefore prospective studies that employ prospective measurement of coital frequency are warranted.

Our study has some limitations. First, we only included 24 women and therefore may be limited in our power to detect differences, particularly smaller effect sizes. The questions that we used for tracking were intentionally kept short to maximize retention, and some have not been validated. For example, we assessed comorbidity using the following question. “Did you feel pain elsewhere in your body?” However, we are limited in assessing the other body pain, as we do not have measures of that pain (e.g., of multiple location, potential origin, or pain intensity at the other sites).

In conclusion, we describe a feasible and acceptable novel method to measure vulvar pain as it varies over time. This method also allows collection of factors that may influence vulvar pain level. Future studies of chronic vulvar pain should evaluate the use of such technology to prospectively assess changes in pain in evaluating the natural history of and treatments for vulvodynia.

## Figures and Tables

**Table 1:** Characteristics of 24 participants with clinically confirmed vulvodynia in Minneapolis-St. Paul, Minnesota, 2013.

Characteristic	*N* = 24 women *N* (%)
Mean age, *y* (SD)	30 (6.2)
Marital status	
Single, never married	15 (63)
Married	7 (29)
Separated/divorced	2 (8)
Education	
High school	1 (4)
Some college	2 (8)
Associate's degree	3 (13)
Bachelor's degree	15 (62)
Graduate degree	3 (13)
Primary vulvodynia	5 (23)
Generalized pain Throughout vestibule	12 (52)
Provoked vulvodynia	10 (42)
Sought care for pain	12 (52)

**Table 2:** Adjusted averages of vulvar pain reports by group for treatment, other pain, pain upon waking, and intercourse.

Group	Adjusted average (SE)		SE for difference	*P* value
	No	Yes		
Treatment	1.50 (0.29)	3.99 (0.45)	0.44	<0.001
Other pain in body	1.87 (0.40)	2.09 (0.43)	0.46	0.64
Pain upon waking	1.63 (0.33)	3.45 (0.46)	0.42	<0.001
Had intercourse	1.76 (0.41)	2.18 (0.42)	0.46	0.36

**Table 3:** Feasibility of smartphone collection for prospective studies of vulvar pain among 23 women who completed one month of weekly tracking of vulvar pain and correlates, Minneapolis-St. Paul, Minnesota, 2013.

Survey questions	*N* (%)
*Experience with tracking health-related issues prior to the study *	
Did you record any of the following prior to the study?	
I did not record any of these items prior to the study	11 (48)
Pain	3 (13)
Communication with doctor	2 (9)
Pain management and/or treatment (medication)	1 (4)
Sexual intercourse	2 (9)
Mood	4 (17)
Pain anywhere else in your body	2 (9)
Diet	4 (17)
Exercise	7 (30)
*Safety of vulvar pain tracking *	
Did recording your vulvar pain make it…?	
A lot worse	0 (0)
Worse	1 (4)
No change	20 (87)
Better	2 (9)
A lot better	0 (0)
How concerned are you that tracking your vulvar pain will make your pain worse?	
Very concerned	0 (0)
Concerned	1 (4)
Somewhat concerned	5 (22)
Neither concerned nor unconcerned	5 (22)
Somewhat unconcerned	2 (9)
Unconcerned	3 (13)
Very unconcerned	7 (30)
*Feasibility of tracking with smartphone *	
How would you rate the ease of answering the questions on your smartphone?	
Very difficult	0 (0)
Difficult	0 (0)
Easy	0 (0)
Very easy	23 (100)
How easy was it for you to perform the tasks of the study?	
Very difficult	0 (0)
Difficult	0 (0)
Neutral	0 (0)
Easy	1 (4)
Very easy	22 (96)
How much did you like the prompts reminding you to take your survey?	
Disliked extremely	0 (0)
Disliked very much	0 (0)
Neither liked nor disliked	2 (9)
Liked very much	7 (30)
Liked extremely	13 (57)
Prefer not to answer	1 (4)
If it was an option, would you be willing to continue recording your vulvar pain over an extended period of time?	
Yes	23 (100)
No	0 (0)
For us to better understand your pain, would you be willing to record your pain level daily instead of weekly?	
Yes	20 (87)
No	3 (13)
During the study, did you find it valuable to record these items? Endorsed responses listed.	
Pain	15 (65)
Pain management and/or treatment	11 (48)
Sexual intercourse	12 (52)
Mood	10 (43)
Pain anywhere else in your body	7 (30)
I did not find it valuable to record any of these items	5 (22)
Prefer not to answer	1 (4)
What factors may reduce your ability to complete a survey within 24-hour prompt? Endorsed responses listed.	
Too busy	8 (35)
Problem(s) with phone	11 (48)
Family responsibilities	1 (4)
Lack of privacy	3 (13)
Boredom with tracking	0 (0)
Out of town or on vacation	8 (35)
Pain prohibits daily activities	0 (0)
Other, please specify	2 (9)
No factors would reduce my ability to complete on time	7 (30)
*Smartphone versus another method *	
Are you more likely to track your vulvar pain using your smartphone versus a computer?	
Much more likely	15 (65)
More likely	5 (22)
Neither more nor less likely	2 (9)
Less likely	1 (4)
Much less likely	0 (0)
Are you more likely to track your pain using your smartphone versus a pen and paper or diary?	
Much more likely	18 (78)
More likely	4 (17)
Neither more nor less likely	0 (0)
Less likely	1 (4)
Much less likely	0 (0)
